# Integrated morphological and biochemical analysis of selected sesame (*Sesamum* spp.) species

**DOI:** 10.3389/fpls.2025.1571363

**Published:** 2025-07-10

**Authors:** Varadha Gayathri V. P., Lovely B., Arya K., Atul Jayapal, Pratheesh P. Gopinath

**Affiliations:** ^1^ Department of Genetics and Plant Breeding, College of Agriculture, Thiruvananthapuram, Kerala, India; ^2^ Department of Agronomy, College of Agriculture, Thiruvananthapuram, Kerala, India; ^3^ Department of Agricultural Statistics, College of Agriculture, Thiruvananthapuram, Kerala, India

**Keywords:** *Sesamum indicum*, *Sesamum radiatum*, *Sesamum mulayanum*, *Sesamum malabaricum*, *morphology*, *biochemical*, *phenol content*

## Abstract

The study aimed to characterize wild relatives and traditional cultivars of sesame based on their morphology and biochemical properties, including crude protein, oil content, fatty acid profiles, and total phenol content. The study included accessions of *Sesamum indicum*, *S. radiatum*, *S. mulayanum*, and *S. malabaricum*. A comparative analysis revealed significant variation in flower color among the species. *S. indicum* had pale purplish-pink flowers, while *S. mulayanum* and *S. malabaricum* had dark violet flowers, and *S. radiatum* had white flowers with violet borders. Differences in capsule hairiness were also noted, with cultivated species having glabrous capsules, while wild species exhibited hairy capsules. The hairiness ranged from weakly hairy capsules in *S. mulayanum* and *S. malabaricum* to strongly hairy capsules in *S. radiatum*. All 27 genotypes produced a single flower per axil, a trait common across all sesame species. The *S. indicum* accession, Ayali 1, produced the most capsules per plant, while *S. radiatum* (IC 256273) produced the fewest. *S. radiatum* accessions had the longest and broadest capsules, while the shortest and narrowest capsules were found in *S. malabaricum*. Cultivated sesame varieties produced larger and heavier seeds compared to wild species. The highest phenol content was recorded in *S. radiatum* (IC 210433), while the lowest was observed in Kayamkulam 1. Seed yield per plant showed a strong positive correlation with the number of capsules per plant, 1000 seed weight, and oil content, while a significant negative correlation was found between phenol content, plant height, and seed yield. Oil content analysis revealed that the highest oil yield came from *Thilak* seeds, while the lowest yield was observed in *S. malabaricum* (IC 557243). Fatty acid profiling showed the presence of both saturated fatty acids (palmitic acid, stearic acid, behenic acid, margaric acid, and arachidic acid) and unsaturated fatty acids (oleic acid, linoleic acid, linolenic acid, eicosanoic acid, 11-eicosenoic acid, and linolelaidic acid) in varying proportions across sesame samples.

## Introduction

Sesame (Sesamum) belongs to the Pedaliaceae family, which comprises 16 genera and 60 species. Out of these, 37 species are classified under *Sesamum*. In India there are about nine taxa of sesame of which one is widely under cultivation and the rest are wild or naturalized. In Kerala six species of sesame are found viz. *S. indicum* (2n=26), *S. malabaricum* (2n=26), *S. mulayanum* (2n=26), *S. radiatum* (2n=64), *S. laciniatum* (2n=26) and *S. prostratum* (2n=32). *S. malabaricum* or *S. indicum* subsp. *malabaricum*, is considered the wild progenitor of cultivated sesame. Both cultivated sesame and *S. malabaricum* are diploid with a chromosome number of 2n=2x=26 and produce fertile offspring when crossed. This genetic evidence supports the idea that *S. malabaricum* is the progenitor of cultivated sesame, unlike other sesame species (Bedigian, 2014). The progeny derived from the cross between *S. malabaricum* and the cultivated sesame exhibited high fertility rate under natural growth conditions (Nimmakayala et al., 2011). It is also reported to have resistance to phyllody disease and hence can be used as a donor parent in resistant breeding programmes (Saravanan and Nadarajan, 2005).

*S. mulayanum* is sometimes seen in sesame crop field as an associated weed (Tanesaka et al., 2012). This species shows deep seed dormancy and is characterized by conspicuous purple pigmentation on the lower lip of the corolla. The seed dormancy of *S. mulayanum* may be due to its seed coat structure (coat-enhanced dormancy). The character purple pigmentation of the lower lip of corolla was found to be linked with the major gene controlling seed dormancy. Tarihal et al. (2003) reported absence of any fertilization barriers in crosses between *S. indicum* and *S. mulayanum* with the latter being the female parent. The flowers of the F1 plants were characterized by pansy violet corolla color (intermediate between the parents). Based on their studies Biswas and Mitra (1990) opined that *S. indicum* and *S. mulayanum* are closely related. The seeds of *S. indicum* and F_1_ of interspecific cross *S. indicum* X *S. mulayanum* showed good germination also.

The center of origin of *S. radiatum* is west tropical Africa. This species was introduced into India as a part of breeding programmes in 1933 at Tamil Nadu Agricultural University, Coimbatore (John and Rao, 1941). It is cultivated and consumed as a leafy vegetable in many parts of the world (Adéoti et al., 2012). The leaves of *S. radiatum* are known to have phenols, antioxidant properties and minerals (Oduntan et al., 2011). It has stronger tap root with more stout fibrous roots. Correspondingly, *S. radiatum* has higher tolerance to biotic and abiotic stresses, compared with the cultivated sesame and some other wild species. *S. laciniatum* is perennial, prostrate, profusely branched, having deeply dissected coarse leaves with deep purple flowers with purple anthers where yellow glands absent, small laterally compressed tough capsules and deeply reticulated seeds with thick testa (Salunke et al., 2023).

*S. indicum* is the only cultivated species representing the sesame germplasm (Venkataramana Bhat et al., 1999). Sesame cultivation dates back to the ancient Indus Valley Civilization, making it one of the earliest cultivated crops. These small seeds, often referred to as the “seeds of immortality,” contain 44-58% oil, 18-25% protein, 13.5% carbohydrates, and 5% ash (Borchani et al., 2010). Sesame seeds are a rich source of energy, and they are packed with vitamins E, A, B complex, and minerals such as calcium, phosphorus, iron, copper, magnesium, zinc, and potassium. They are also an excellent substitute for mother’s milk, especially in cases of milk allergies (Ranganatha et al., 2010). Sesame oil, due to its nourishing, calming, and warming properties, is widely used as the base in over 90% of Ayurvedic treatments, making it an ideal massage oil (Shamloo et al., 2019; Biswas et al., 2018).

Sesame is a highly valued crop worldwide because of its various uses for its seeds and oil in the food, nutraceutical, and pharmaceutical industries. Global sesame seed production exceeded 7 million tons in 2022, with Africa and Asia producing over 95% of the world’s supply of sesame. In 2022, the highest producing countries were Sudan, India, Myanmar, the United Republic of Tanzania, and China, accounting for over 60% of global production (Anyogu et al., 2024).

Known as “gingelly” in some regions, *S. indicum* is referred to as the “queen of oilseed crops” (Biswas et al., 2018) and is traditionally cultivated in the Onattukara region of Kerala, India. This region is particularly famous for the quality of its sesame seeds and oil, grown in uplands and summer rice fallows. Three species of wild sesame—*S. malabaricum*, *S. mulayanum* and *S. radiatum*—are distributed throughout the Onattukara region, suggesting it may be a secondary center of origin for sesame. A preliminary study on the morphological and biochemical traits of sesame collections from this region was attempted which indicates significant variation and considerable genetic diversity. Documenting these characteristics is crucial for the conservation of sesame. The study aims to validate the genetic diversity and assess the therapeutic and agronomic potential of sesame in the Onattukara region.

## Materials and methods

The study involved accessions of the widely distributed *Sesamum* species in the sesame growing tract of Onattukara region. Six accessions of *S. radiatum*, seven accessions of *S. mulayanum*, five accessions of *S. malabaricum*, five accessions of the traditional sesame cultivar *Ayali*, and four cultivated sesame varieties from the Onattukara region were evaluated in the study. The seeds of these 27 genotypes were sown in a randomized block design with three replications in January 2022 with rows spaced at 30 cm x 30 cm. The experiment was conducted at the Onattukara Regional Agricultural Research Station, Kayamkulam, under the Kerala Agricultural University. The station is located in the Onattukara tract, which lies between 8°55′44″ to 9°21′09″ N latitude and 76°23′13″ to 76°41′16″ E longitude.

Onattukara is a region characterized by a humid tropical climate. The predominant soil type is loamy sand, comprising 83–89% sand, 5% silt, and 5.8% clay, with a field capacity of 16.05%. The soil is porous, acidic in nature, and has low organic matter and nutrient content. Nutrient levels include 0.145% nitrogen, 0.121% phosphorus, 0.0185% potassium, 0.098% calcium, and 0.035% magnesium. Due to its low water-holding capacity, the region faces waterlogging issues during the rainy season and drought conditions during the summer. Temperatures are higher from January to April and lower between June and September. The region experiences a moderate climate, with mean minimum temperatures ranging from 22.0°C to 25.2°C and mean maximum temperatures ranging from 29.0°C to 32.9°C. Relative humidity is higher in the morning and lower in the evening, ranging between 70.0% and 80.1%. Monthly rainfall in the Onattukara region varies widely, from 21.5 cm to 473.2 cm.

### Pre-treatment of seeds

Both *S. radiatum* and *S. mulayanum* are wild sesame species that exhibit innate dormancy, resulting in poor germination. To overcome this, seeds were pretreated with gibberellic acid (GA3). Specifically, the seeds were soaked in a 300 ppm solution of GA3 for 18 hours, and then they were sown in the field.

### Field management

All intercultural operations were conducted according to the Package of Practices recommendations of Kerala Agricultural University (KAU, 2016). Wild cultivars of the same species were planted together, maintaining a spacing of 30 cm x 30 cm, with an alleyway of 60 cm left bare between cultivars from different species. Fertilization was done at the time of land preparation using a 30:15:30 kg/ha NPK ratio. Thinning and earthing-up were performed 15 days after sowing, while weeding was done at 15 and 30 days after sowing (DAS).

### Morphological and quality parameter measurements

Biometric data on various morphological parameters were recorded from five randomly selected plants of each genotype, following standard descriptors (IPGRI and NBPGR, 2004. and protocols. Quality parameters relevant to sesame, including oil content, crude protein content, fatty acid profiling, and total phenol content, were analyzed from the seeds as described below:

### Oil content (%)

Oil was extracted from sesame seeds using a cold hexane extraction method. A 1-gram sample of dried and ground sesame seeds was placed in a thimble and subjected to hexane extraction in a Soxhlet apparatus for 6 hours. The hexane was then removed under reduced pressure using a rotary evaporator, and the oil content was calculated following the method described by Latif and Anwar (2011).

### Crude protein content (%)

Crude protein content was estimated using the Bradford method. Sesame seeds were ground and extracted (1:5, w/v) overnight in 4% sodium bicarbonate at 4°C. The extracts were centrifuged, filtered, and the protein content was measured using serum albumin as a standard (Leduc et al., 2006).

### Fatty acid profiling

For fatty acid profiling, 50 mg of sesame oil was placed in 50 ml screw-capped Pyrex tubes (50 cm length, 1 cm internal diameter). To each tube, 2 ml of methanolic sulfuric acid was added, and the tubes were heated in an oil bath at 80°C for 2 hours, with stirring. After cooling, 2 ml of distilled water were added to stop the reaction. The esterified fatty acids were extracted twice with HPLC-grade hexane. The hexane layer was washed with 2% sodium bicarbonate, dried over sodium sulfate, and stored at -20°C before analysis by GC-MS to obtain the fatty acid profile (IUPAC, 1987).

GC-MS analysis of the oil extracted from the seeds was performed using the equipment Shimadzu GC-20101, Kyoto, Japan. The column used was Stabilwax GC Capillary Column with dimensions of 60 mm, ID × 0.25 μm film. The carrier gas used is Helium with at low of 1.0 ml/min. The oven temperature was programmed as follows: 70°C initial temperature, then gradually increased to 140°C at 10°C/min and to 240°C @3°C per min. The identification of components was based on NIST17 libraries.

### Total phenol content

One gram of sesame seeds was ground and soaked in methanol, stirred at 200 rpm for 18 hours. The methanolic extract was then analyzed for total phenol content using the Folin-Ciocalteau reagent. Phenols react with phosphomolybdic acid in an alkaline medium to form a blue-colored complex (Meda et al., 2005).

### Fatty acid composition and oil stability

The total saturated and unsaturated fatty acid contents were determined directly from the GC chromatogram as percentages of the respective fatty acids. Data from the GC analysis were used to compute the Polyunsaturated Fatty Acid (PUFA) content, Oleic Desaturation Ratio (ODR), and Linoleic Desaturation Ratio (LDR) using the equations from Pleines and Friedt (1988) and Fatemi and Hammond (1980).

### Oil composition

The oil composition refers to the ratio of oleic to linoleic acid. A higher oil composition ratio indicates better oxidative stability of the oil, which is a key factor for evaluating the quality and longevity of sesame oil.

#### Oil stability

The oil stability is indicated by two parameters *viz.*, oleic desaturation ratio (ODR) and linoleic desaturation ratio (LDR). It is calculated as follows:


$$\text{ODR}=\frac{\text{Linoleicacid }(\%)+\text{Linolenic acid }(\%)}{\text{Oleic acid }(\%)+\text{Linoleic acid }(\%)+\text{Linolenic acid }(\%)}$$

$$\text{LDR}=\frac{\text{Linolenic acid }(\%)}{\text{Linoleic acid }(\%)+\text{Linolenic acid }(\%)}$$

#### PUFA

Poly unsaturated fatty acid composition can be calculated as follows:


PUFA=Linoleic acid (%)+Linolenic acid (%)

### Statistical analysis

The data were subjected to analysis of variance (ANOVA) and various genetic parameters were worked out using GRAPES software, version 1.1.0 (Gopinath et al., 2020).

## Results and discussion

### Morphological characters

**Figure 1:**
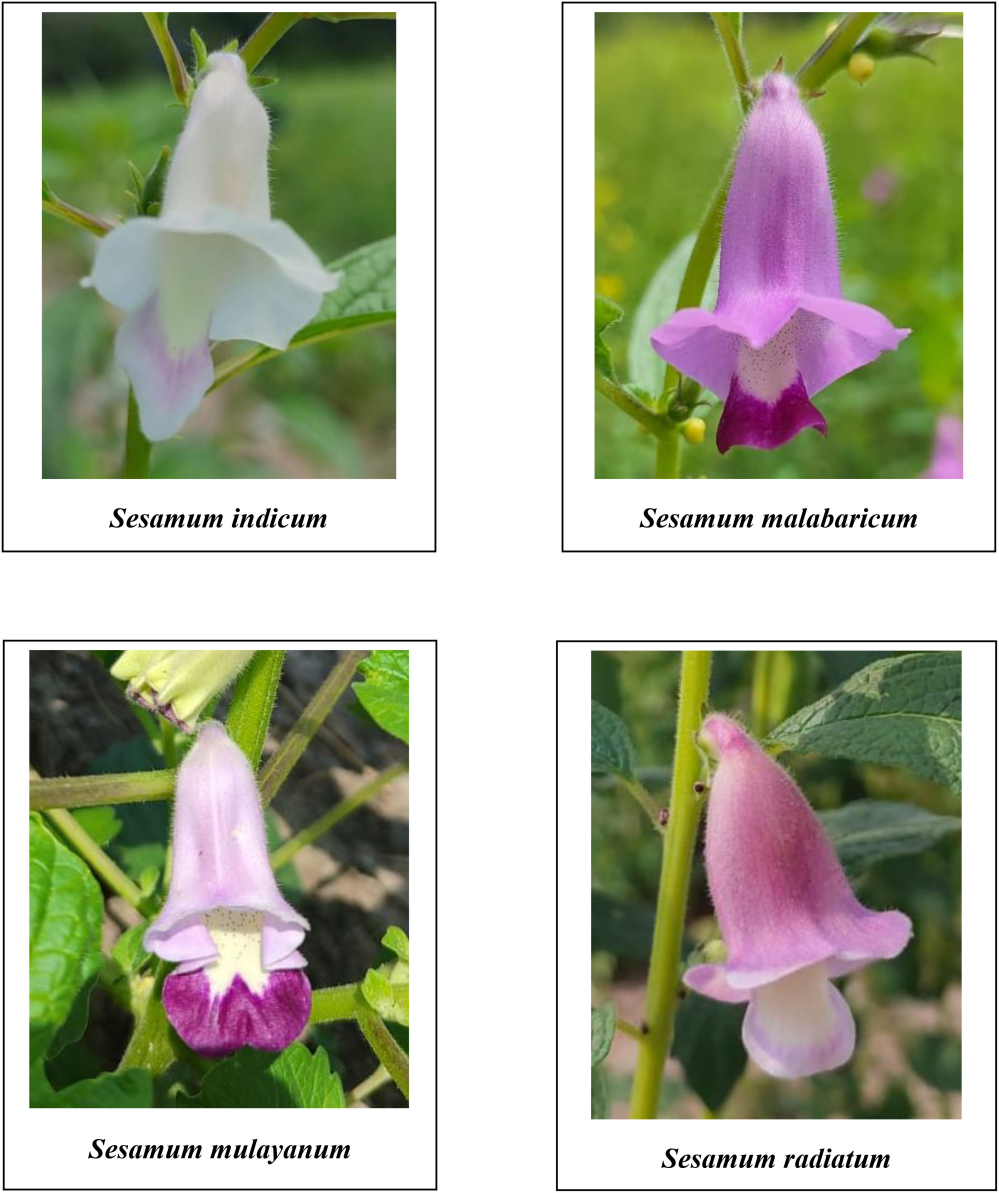
Exterior corolla of the flowers of *Sesamum* species in the study.

**Figure 2:**
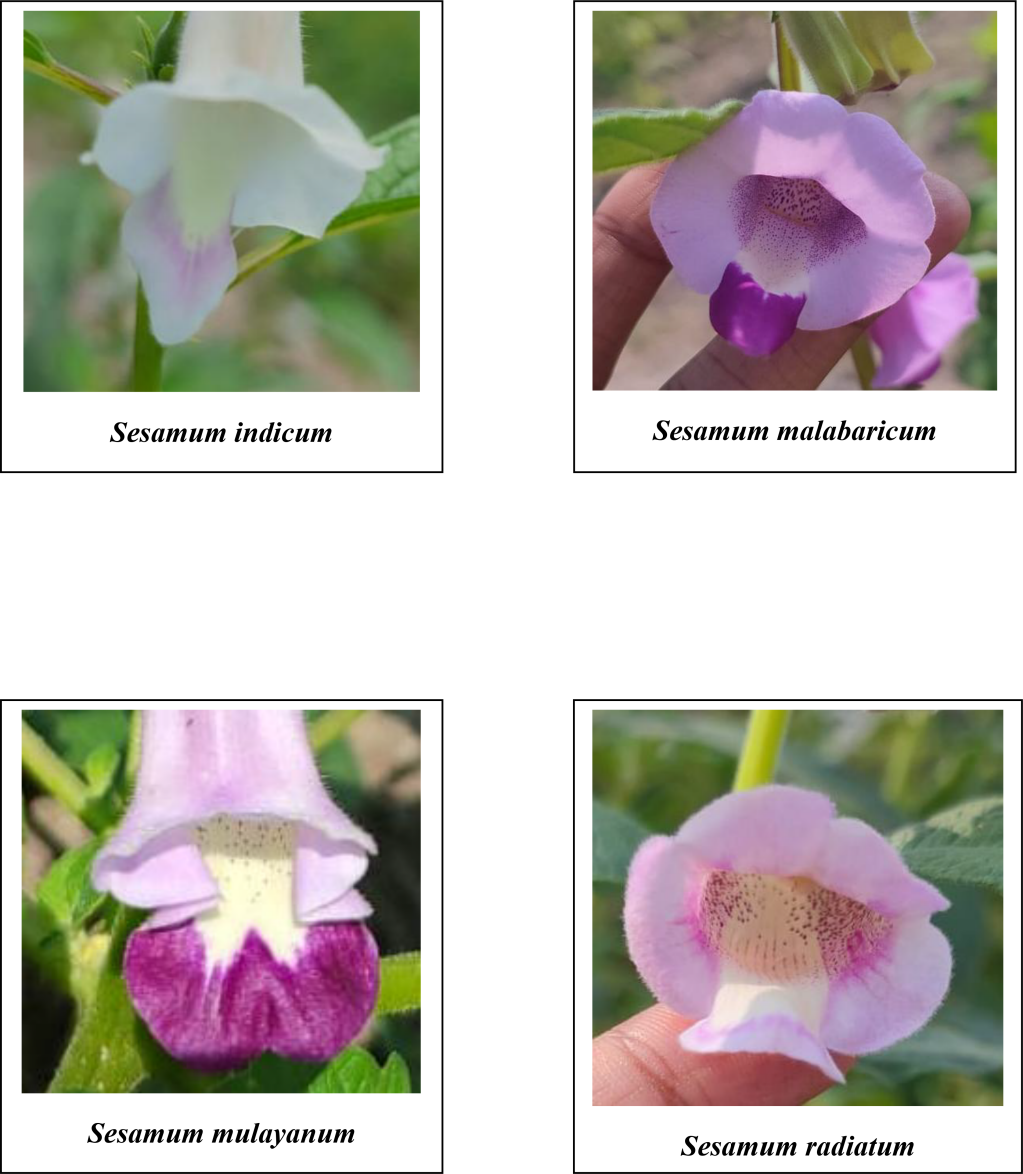
Exterior corolla of the flowers of *Sesamum* species in the study.

**Figure 3:**
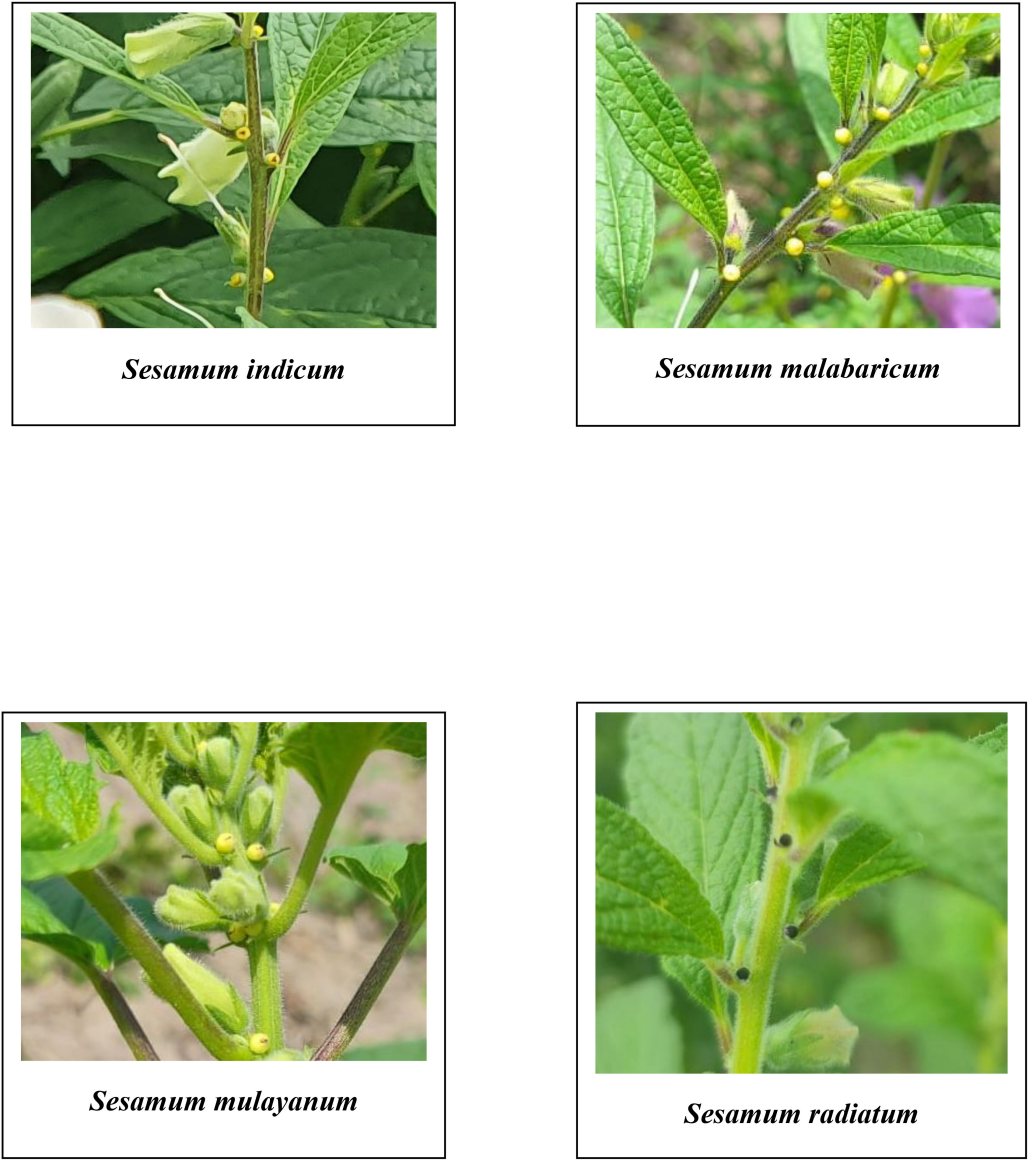
Extra floral nectaries of *Sesamum* species in the study.

**Figure 4:**
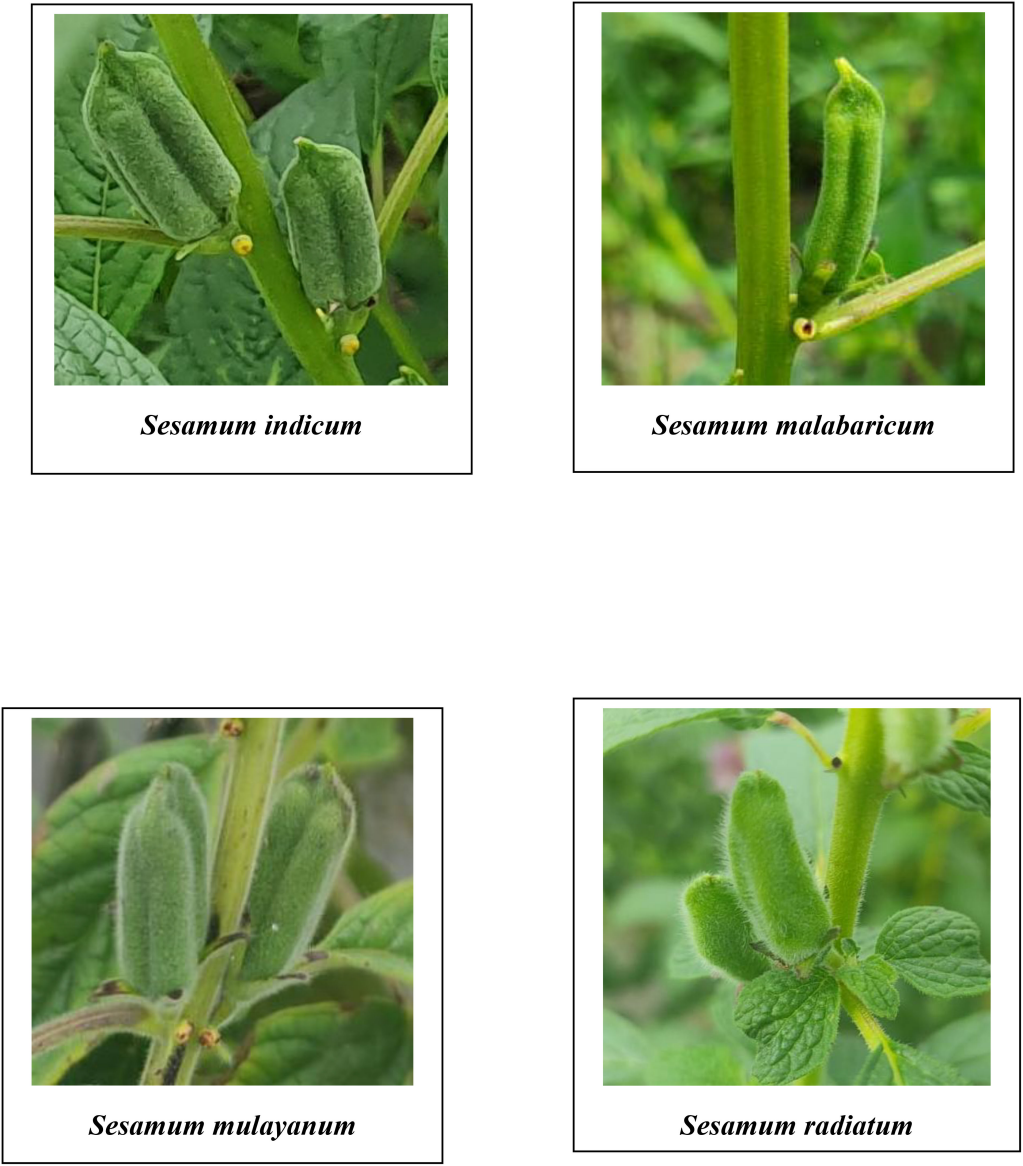
Capsules of *Sesamum* species in the study.

**figure 5:**
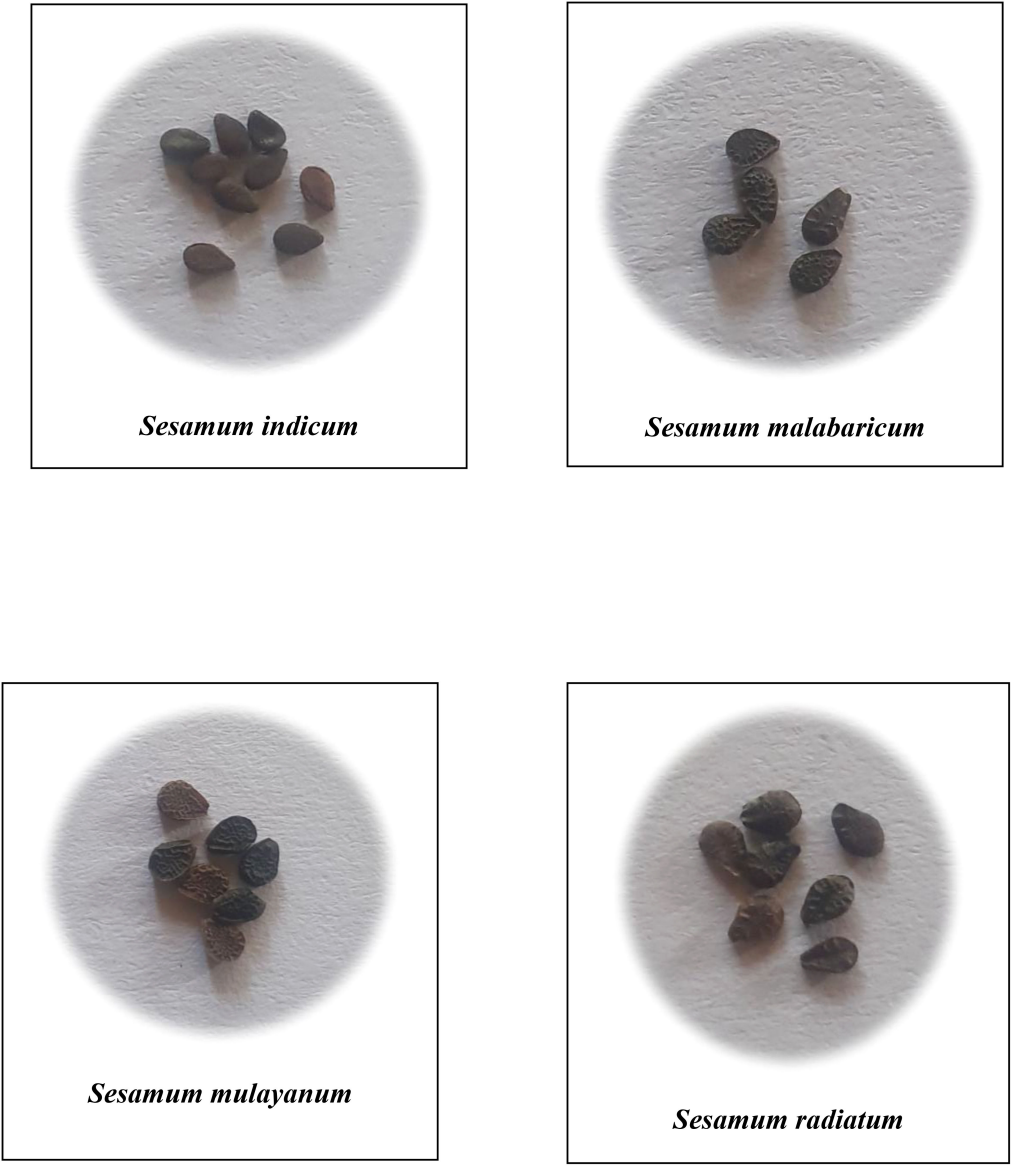
Seeds of *Sesamum* species in the study.

A comprehensive analysis of qualitative morphological traits, such as plant growth type, exterior corolla color, interior corolla color, lower lip color, capsule hairiness, and seed coat color, revealed considerable variation across 27 genotypes from four different species (**Figures 1**–**5**). These characters displayed significant variation between cultivated varieties, the traditional cultivar Ayali, and wild relatives (**Table 1**).

**Table 1:** Differences between accessions of *Sesamum* species with respect to morphological characters.

Sl. No.	Species	Accession number/Variety	Plant growth type	Exterior corolla colour	Interior corolla colour	Lower lip colour	Capsule hairiness	Seed coat colour
1.	*Sesamum indicum*	Kayamkulam 1	Indeterminate	White with pink shading	White	Pale purplish pink	Glaborous	Brown
2.		Thilak						
3.		Thilathara						
4.		Thilarani						
5.		Ayali 1						
6.		Ayali 2						
7.		Ayali 4						
8.		Ayali 5						
9.		Ayali 11						
10.	*Sesamum mulayanam*	IC 557231		Light violet		Dark violet	Weak	Black
11.		IC 277406						
12.		IC 277417-X						
13.		IC 557232						
14.		IC 199447						
15.		SML 1						
16.		SML 2						
17.	*Sesamum malabaricum*	IC 557251		Light violet				Brown
18.		IC 621506						
19.		IC 557244						
20.		IC 623409						
21.		IC 557243						
22.	*Sesamum radiatum*	IC 210433		Dark violet		White with light violet border	Strong	Black
23.		IC 623402						
24.		IC 208681						
25.		IC 256273		Pink				
26.		IC 208663		Dark violet				
27.		SR 1						

The plant growth type of both cultivated and wild genotypes, including the traditional cultivar Ayali, exhibited a common indeterminate growth habit. All genotypes showed a prolonged flowering stage, non-synchronous capsule ripening, and non-synchronous capsule shattering. This indeterminate growth habit aligns with findings by Kumari and Ganesamurthy (2015) and Tesfaye et al. (2021), who also reported similar growth characteristics in sesame.

The exterior corolla color displayed a wide spectrum, ranging from white to dark violet. Cultivated varieties and the traditional Ayali cultivar had white corollas with pink shading, while the wild varieties exhibited hues from light violet to dark violet, with varying shades within each species. Specifically, *S. malabaricum* and *S. mulayanum* showed light violet exterior corolla colors, with some variation in shade. In contrast, *S. radiatum* stood out with a distinct dark violet exterior corolla color, also showing variation in its shading. This observation is consistent with Kumari and Ganesamurthy (2015), who noted violet shades in wild sesame species and white shades in cultivated varieties. Tesfaye et al. (2021) documented that 6.78% of cultivated sesame in Ethiopia had a white corolla, 70.85% had a white corolla with pink shading, and 22.37% had a white corolla with deep pink shading.

The interior corolla color was uniformly white across all genotypes, with variations only in the degree of pigmentation. Cultivated and traditional varieties exhibited light pigmentation, while wild relatives displayed deeper pigmentation in the interior corolla. These findings align with those of Tesfaye et al. (2021), who reported similar pigmentation patterns in *S. indicum*.

The lower lip color of the flower varied across species. In *S. radiatum*, the lower lip was white with a violet border, while in *S. malabaricum* and *S. mulayanum*, it appeared as dark purplish-pink. For cultivated and traditional varieties, the lower lip was pale purplish-pink. Regarding capsule hairiness, *S. radiatum* exhibited profuse hairiness, whereas both *S. malabaricum* and *S. mulayanum* had weak capsule hairiness. In contrast, the capsules of both traditional and cultivated varieties were glabrous. These results are consistent with previous studies by Kumari and Ganesamurthy (2015) and Tesfaye et al. (2021), who observed similar trends in capsule hairiness and lower lip color.

Seed coat color varied from brown to black. *S. malabaricum*, traditional, and cultivated varieties produced brown seeds, while *S. radiatum* consistently yielded black seeds. Kumari and Ganesamurthy (2015) reported dull black seeds for *S. malabaricum*, bright black seeds for *S. radiatum*, and seeds with varying colors for *S. indicum*.

### Biometric characters

**Figure 6:**
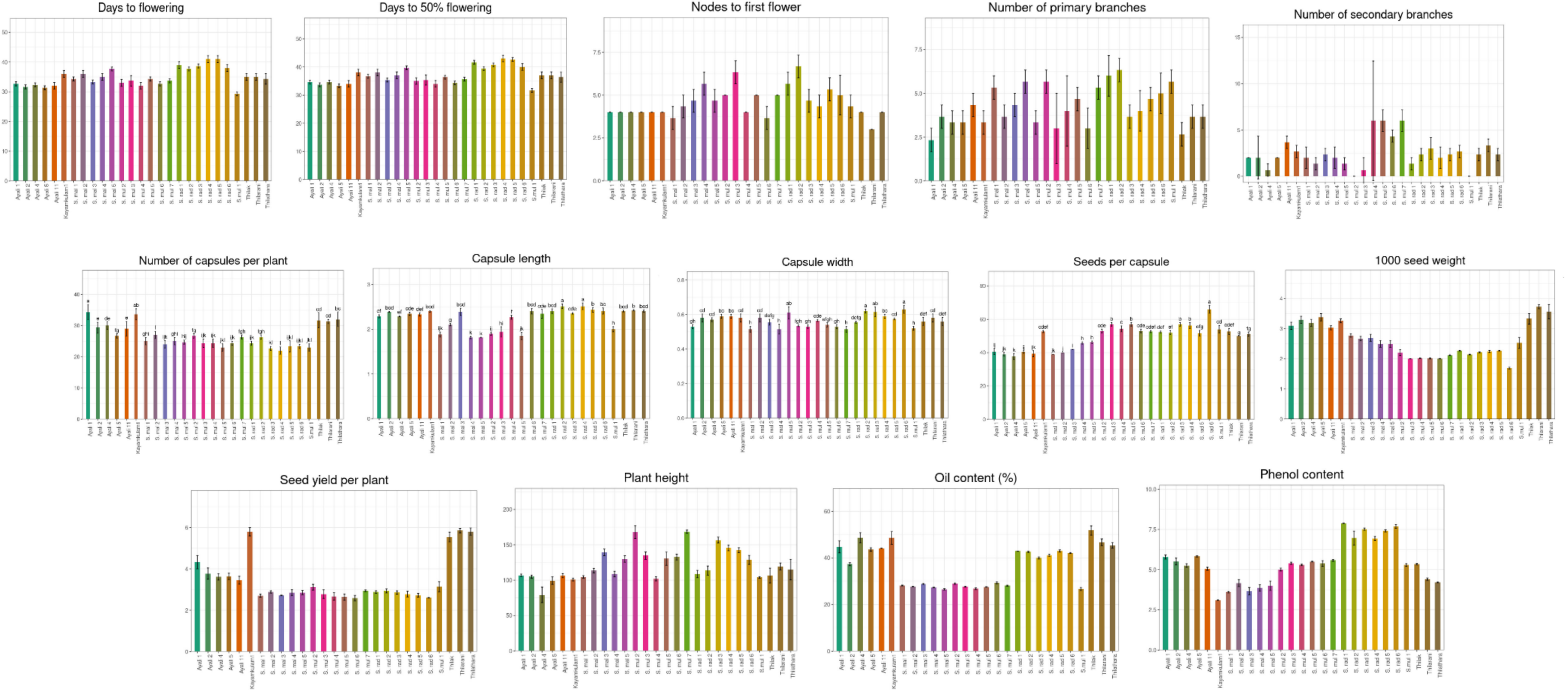
Variation among *Sesamum* species with respect to biometric characters.

The biometric parameters of various *Sesamum* accessions were recorded, and their mean values are presented in **Table 2**. All traits exhibited substantial variation among the genotypes, indicating a broad genetic base (**Figure 6**).

**Table 2:** Differences between accessions of *Sesamum* species with respect to biometric characters.

Sl. No.	Species	Accession number/Variety	Days to flower initiation	Days to 50% flowering	No. of nodes to first flower	No. of 1^0^ branches	No. of 2^0^ branches	No. of capsules/ plant	Plant height (cm)
1.	*Sesamum indicum*	Kayamkulam 1	36.000	38.000	4.000	3.333	2.667	33.667	101.000
2.		Thilak	35.000	37.000	4.000	2.667	2.333	31.667	106.467
3.		Thilathara	34.333	36.333	4.000	3.667	2.333	32.000	115.000
4.		Thilarani	35.000	37.000	3.000	3.667	3.333	31.333	119.333
5.		Ayali 1	32.667	34.667	4.000	2.333	2.000	34.333	106.933
6.		Ayali 2	31.500	33.667	4.000	2.333	2.000	29.333	105.000
7.		Ayali 4	32.333	34.667	4.000	3.667	0.667	30.000	79.333
8.		Ayali 5	31.333	33.333	4.000	3.333	2.000	26.667	99.333
9.		Ayali 11	32.000	34.000	4.000	3.333	3.667	29.000	106.333
10.	*Sesamum mulayanam*	IC 557231	29.333	31.667	4.333	4.333	0.000	23.000	103.933
11.		IC 277406	33.000	35.000	5.000	5.667	0.000	26.667	167.833
12.		IC 277417-X	33.667	35.333	6.333	5.667	0.667	24.333	134.667
13.		IC 557232	32.000	34.000	4.000	3.000	6.000	24.333	102.067
14.		IC 199447	34.333	36.333	5.000	4.000	6.000	23.000	130.333
15.		SML 1	32.667	34.333	3.667	4.667	4.333	24.333	132.667
16.		SML 2	33.667	35.667	5.000	3.000	6.000	26.333	168.667
17.	*Sesamum malabaricum*	IC 557251	34.333	36.667	3.667	5.333	2.000	25.000	104.667
18.		IC 621506	36.000	38.000	4.333	3.667	1.333	27.000	113.533
19.		IC 557244	33.333	35.333	4.667	4.333	2.333	24.000	139.333
20.		IC 623409	35.000	37.000	5.667	5.667	2.000	25.000	108.667
21.		IC 557243	37.667	39.667	4.667	3.333	1.333	24.667	130.000
22.	*Sesamum radiatum*	IC 210433	39.000	41.667	5.667	6.000	1.333	24.333	108.667
23.		IC 623402	37.667	39.333	6.667	6.333	2.333	26.333	113.667
24.		IC 208681	38.667	40.667	4.667	3.667	3.000	22.667	156.667
25.		IC 256273	41.000	43.000	4.333	4.000	2.000	22.000	145.667
26.		IC 208663	41.000	42.667	5.333	4.667	2.333	23.333	142.333
27.		SR 1	38.000	40.000	5.000	5.000	2.667	23.333	128.667
	Mean		34.8333	36.8519	4.5556	4.2098	2.4691	26.5802	121.1395
	CD (5%)		1.467	1.421	0.739	1.301	2.181	1.883	6.635
	SE(d)		0.731	0.708	0.369	0.648	1.087	0.939	3.307

Sl. No.	Species	Accession number/Variety	Mean capsule length (cm)	Mean capsule width (cm)	Seeds per capsule	1000 seed weight (g)	Seed yield per plant (g)	Oil content (%)	Total phenol content
1.	*Sesamum indicum*	Kayamkulam 1	2.403	0.580	52.667	3.267	5.789	48.560	3.105
2.		Thilak	2.407	0.560	52.667	3.333	5.548	51.765	5.345
3.		Thilathara	2.407	0.560	51.000	3.567	5.807	45.400	4.210
4.		Thilarani	2.430	0.580	50.000	3.733	5.848	46.695	4.400
5.		Ayali 1	2.290	0.530	40.667	3.100	4.325	44.720	5.780
6.		Ayali 2	2.390	0.580	39.000	3.300	3.775	37.285	5.510
7.		Ayali 4	2.290	0.570	37.667	3.200	3.613	48.615	5.255
8.		Ayali 5	2.347	0.590	40.333	3.383	3.639	43.595	5.820
9.		Ayali 11	2.333	0.590	39.333	3.033	3.455	44.145	5.060
10.	*Sesamum mulayanam*	IC 557231	2.000	0.520	54.000	2.530	3.140	26.670	5.290
11.		IC 277406	1.900	0.535	53.000	2.207	3.118	28.935	5.010
12.		IC 277417-X	1.945	0.530	57.000	2.003	2.780	27.625	5.395
13.		IC 557232	2.270	0.565	54.333	2.020	2.671	26.795	5.295
14.		IC 199447	1.850	0.540	57.000	2.013	2.640	27.565	5.500
15.		SML 1	2.405	0.530	53.000	2.007	2.589	29.300	5.380
16.		SML 2	2.345	0.515	52.667	2.207	2.944	28.075	5.580
17.	*Sesamum malabaricum*	IC 557251	1.885	0.515	39.000	2.777	2.706	28.151	3.610
18.		IC 621506	2.105	0.580	40.000	2.673	2.886	27.725	4.165
19.		IC 557244	2.390	0.555	42.000	2.700	2.719	28.815	3.660
20.		IC 623409	1.815	0.515	45.667	2.500	2.853	27.320	3.860
21.		IC 557243	1.815	0.610	46.333	2.500	2.856	26.545	3.995
22.	*Sesamum radiatum*	IC 210433	2.405	0.555	52.333	2.263	2.882	42.865	7.880
23.		IC 623402	2.515	0.620	52.000	2.143	2.935	42.555	6.960
24.		IC 208681	2.355	0.615	57.000	2.213	2.859	39.960	7.520
25.		IC 256273	2.515	0.590	56.333	2.247	2.783	41.165	6.940
26.		IC 208663	2.435	0.575	51.667	2.267	2.729	42.975	7.415
27.		SR 1	2.410	0.630	66.000	1.693	2.606	42.130	7.685
	Mean		2.2466	0.5643	49.3580	2.6220	3.4257	36.8871	5.3935
	CD (5%)		0.074	0.026	1.877	0.139	0.215	1.488	0.215
	SE(d)		0.037	0.013	0.936	0.069	0.107	0.742	0.107

Among the four sesame species studied, late flower initiation was observed in *S. radiatum* accessions IC 256273 and IC 208663, with flowering occurring at 41 days after sowing. Dansi et al. (2012) recorded flowering times of 56.75 and 46.33 days for *S. radiatum* accessions in their study. In contrast, early flowering was observed in *S. mulayanum* accession IC 557231, which flowered at 29 days. The average number of days to 50% flowering for the 27 genotypes was 36.85, closely aligning with Kalaiyarasi et al. (2019), who recorded an average of 35.40 days to 50% flowering. The latest 50% flowering occurred in *S. radiatum* accession IC 256273 at 43 days, while *S. mulayanum* accession IC 557231 reached 50% flowering at 31.67 days. The observed variation in flowering times is likely due to genetic diversity among the accessions, which belong to different species. Similar variations in flowering times have been reported by Hukumchand and Parameshwarappa (2020); Teklu et al. (2021); Kumar et al. (2022), and Gedifew et al. (2023). Within-species variation may be attributed to both environmental factors and genetic differences among accessions.

All 27 sesame genotypes produced a single flower per axil, indicating that this trait is universal across all sesame species. The average number of nodes to first flower across the 27 genotypes was 4.56. The first flower was located farthest from the base in *S. radiatum* accession IC 623402, while it was closest to the base in the traditional cultivar Thilarani. The variation in the number of nodes to first flower is likely a result of genetic differences among the species.

The number of primary branches varied widely among the 27 genotypes, with an average of 4.21 branches. The maximum number of primary branches was observed in *S. radiatum* accession IC 623402, while the fewest primary branches were produced by the traditional cultivar Ayali 1. Other accessions, including *S. radiatum* (IC 210433), *S. mulayanum* (IC 557231, IC 277406, SML 2), and *S. malabaricum* (IC 557251, IC 623409), had a comparable number of primary branches to IC 623402. In contrast, Thilak, Ayali 5, Ayali 4, Kayamkulam 1, *S. malabaricum* (IC 557243), and *S. mulayanum* (IC 277417-X, SML 1) were on par with Ayali 1. Secondary branch numbers were higher in *S. mulayanum* (IC 557232, IC 199447, SML 2), while IC 557231 and IC 277406 (*S. mulayanum*) did not produce secondary branches. The variation in the number of branches can be attributed to genetic differences between genotypes and environmental influences. Teklu et al. (2021) reported a mean of 4 primary branches and 2 secondary branches, and Rahna et al. (2023) found minimal environmental impact on branch numbers in sesame.

*S. mulayanum* accessions were the tallest among the four sesame species, with Ayali 4 being the shortest. The variation in plant height can be attributed to both genetic and environmental influences, in line with findings by Dansi et al. (2012); Kalaiyarasi et al. (2019), and Sultana et al. (2019).

The highest number of capsules per plant was observed in Ayali 1, while the lowest number was observed in *S. radiatum* accession IC 256273. *S. mulayanum* (IC 557231, IC 199447), *S. radiatum* (IC 208681, IC 208663, and SR 1), all produced similar numbers of capsules to IC 256273 of *S. radiatum*. The significant variation in capsule production is likely due to genotypic differences, as similarly noted by Sultana et al. (2019); Kumar et al. (2022), and Akkiligunta et al. (2024). All 27 genotypes exhibited four locules per capsule, a trait consistent across all sesame species.

In terms of capsule morphology, *S. radiatum* accessions produced the longest and broadest capsules, while *S. malabaricum* accessions had the shortest and narrowest capsules. Some *S. mulayanum* accessions were comparable to *S. malabaricum* in capsule dimensions. The variation in capsule length and width is mainly attributed to genetic differences. Dansi et al. (2012) observed capsule length ranging from 2.74 to 3.30 cm and width from 0.7 to 0.8 cm among *S. radiatum* genotypes. Hukumchand and Parameshwarappa (2020) reported capsule length variation between 1.74 and 2.93 cm, and Sultana et al. (2019) found variation between 2.3 and 2.81 cm among different sesame genotypes.

Seeds per capsule averaged 49.36, with the most seeds found in *S. radiatum* accession SR 1, and the least number in Ayali 4. This variation is largely due to genetic differences among genotypes, as confirmed by Saravanan et al. (2020); Manjeet et al. (2020), and Teklu et al. (2021). The heaviest seeds were observed in the cultivated variety Thilarani, while the lightest seeds were found in *S. radiatum* accessions. This variation in 1000-seed weight can be attributed to genetic differences. Overall, cultivated varieties generally had heavier and larger seeds compared to wild species. Similar variations in 1000-seed weight have been reported by Umamaheswari et al. (2019); Hukumchand and Parameshwarappa (2020); Kumar et al. (2022), and Mahla et al. (2024). Thilarani exhibited the highest yield per plant, while *S. mulayanum* accession SML 1 produced the lowest yield. The variation in seed yield per plant is due to both genetic and environmental factors, as reported by Saravanan et al. (2020); Manjeet et al. (2020) and Kumar et al. (2022).

### Biochemical characters

Biochemical parameters, namely, oil content, phenolic compounds, protein levels, and fatty acid profiles were analyzed across the different species and are presented in **Tables 2**–**4**.

**Table 3:** Fatty acid profile of *Sesamum* accessions.

Fatty acids	*S. indicum*								*S. mulayanum*	*S. malabaricum*	*S. radiatum*
	KYM-1	Thilak	Thilathara	Thilarani	Ayali-1	Ayali-2	Ayali-4	Ayali-5			
Oleic acid	43.45	43.05	44.82	43.93	43.12	45.29	42.44	39.06	36.56	35.64	32.91
Linoleiacid	38.96	39.74	37.06	38.8	39.2	37.83	39.09	45.38	40.84	37.93	39
Linolenic acid	0.29	0.23	0.29	0.29	0.3	0.25	0.29	0.32		0.70	0.82
Palmitic acid	9.13	8.81	9.28	9.29	9.27	9.03	9.32	8.46	11.93	13.56	12.56
Palmitoleic acid	0.07	0.07	0.09	0.08	0.08	–	0.11	–			
Steric acid	6.62	6.59	6.95	6.72	6.54	6.44	6.94	6.06	8.31	9.65	11.66
Eicosanoic acid	–	–	–	0.7	–	0.63	0.73	0.69			
11-Eicosenoic acid	0.15	0.1	0.1	0.13	0.14	0.2	0.16	–		0.30	
Behenic acid	0.31	0.11	0.16	0.27	0.18	–	0.2	–		0.38	0.45
Margaric acid	–	–	–	0.07	0.07	–	0.07	–			
Linolelaidic acid	0.21	0.62	0.41	–	0.32	0.33	0.58	–			
Arachidic acid	0.78	0.63	0.71	–	0.71	–	–	–	1.10	1.39	2.13
Oil content (%)	48.560	51.565	45.400	46.695	44.720	37.285	48.615	43.595	27.8521	27.7112	41.9417
Oil Composn	1.1152	1.0833	1.2094	1.1322	1.1000	1.1972	1.0857	0.8607	0.8952	0.9396	0.8438
PUFA	39.25	39.97	37.35	39.09	39.5	38.08	39.38	45.7	40.84	38.63	39.82
ODR	0.4746	0.4815	0.4545	0.4709	0.4781	0.4568	0.4813	0.5392	0.5276	0.5201	0.5475
LDR	0.0074	0.0058	0.0078	0.0074	0.0076	0.0066	0.0074	0.0070	0	0.0181	0.0206

**Table 4:** Mean values of morphological and biochemical characters in *Sesamum* species.

Species	Morphological		Biometrical		Biochemical				
	Lower lip colour of flower	Capsule hairiness	1000 seed weight (g)	Seed yield per plant (g)	Oil content (%)	Total phenol content (mg GAE/g)	Lignins		Protein (g)
							Sesamin (%)	Sesamolin (%)	
*S. indicum*	Pale purplish pink	Glaborous	3.3240	4.6443	45.6422	4.9428	1.5958	0.6818	15.38
*S. mulayanam*	Dark violet	Weak	2.1410	2.8403	27.8521	5.350	–	–	17.7
*S. malabaricum*	Dark violet	Weak	2.6300	2.8040	27.7112	3.8580	2.533	0.485	15.3
*S. radiatum*	White with light violet border	Strong	2.1377	2.7990	41.9417	7.4000	1.581	1.981	16.5

The oil content across the 27 sesame genotypes averaged 36.89%. The highest oil yield was recorded for Thilak, while the lowest was found in *S. malabaricum* (IC 557243). These findings are consistent with the results of Valarmathi et al. (2003); Hiremath et al. (2007) and Akhila and Beevy (2015). Oil yield in *S. malabaricum* (IC 621506, IC 623409) and *S. mulayanum* (IC 557231, IC 277417-X, IC 557232, IC 199447) was similar to IC 557243 of *S. malabaricum*.

Phenolic compounds, such as flavonoids, phenolic acids, and tannins, play a crucial role in stabilizing lipid oxidation and contribute to antioxidant activity. The mean phenol content across the 27 genotypes was 5.39 mg GAE/g. *S. radiatum* (IC 210433) had the highest phenol content, while the lowest was found in Kayamkulam 1. The high phenol content in *S. radiatum* aligns with findings by Pathak et al. (2020) and Oduntan et al. (2011). In contrast, Akhila and Beevy (2015) reported higher phenol content in *S. indicum* oils. The phenol content exhibited minimal environmental variation, as evidenced by the small difference between PCV (24.527) and GCV (24.408), along with high heritability (99%) and genetic advance (50.04%).

### Fatty acid profiling

Fatty acids, major components of plant, animal, and microbial lipids, play a crucial role in oil quality. Oleic and linoleic acids are the predominant fatty acids in sesame oil, comprising approximately 80% of the total oil content. Studies on fatty acid composition in various germplasm collections have revealed significant variation in the proportions of saturated and unsaturated fatty acids, which could potentially lead to the development of higher-quality edible oils.

The fatty acid profiles of four cultivated varieties (KYM 1, Thilak, Thilathara, Thilarani), four traditional varieties (Ayali 1, Ayali 2, Ayali 4, Ayali 5), and wild relatives (*S. malabaricum*, *S. mulayanum*, and *S. radiatum*) were examined. The analysis revealed the presence of both saturated fatty acids (e.g., palmitic acid, stearic acid, behenic acid, margaric acid, and arachidic acid) and unsaturated fatty acids (e.g., oleic acid, linoleic acid, linolenic acid, eicosanoic acid, 11-eicosenoic acid, and linolelaidic acid) in varying proportions across the seed oils of the sesame samples.

Oleic acid, the primary unsaturated fatty acid in sesame oil, was found in the highest amounts in Ayali 2, a traditional cultivar from the Onattukara region. Wild sesame relatives exhibited lower oleic acid content, with *S. radiatum* showing the least amount. These results are consistent with Mondal et al. (2010) and Hiremath et al. (2007). Linoleic acid content was highest in Ayali 5 (45.38%), while Thilathara exhibited the least. Sesame cultivars with higher linoleic acid content, such as Ayali 5, could be valuable for breeding programs aimed at enhancing linoleic acid content in sesame. *S. radiatum* had the highest linolenic acid content, while *S. indicum* showed the lowest.

*S. malabaricum* (13.56%) exhibited the highest palmitic acid content, while Thilak had the lowest. Mondal et al. (2010) reported different results in terms of palmitic acid content. Palmitoleic acid was absent in the oil of wild sesame species, while Ayali 4 contained relatively higher amounts (0.11%). Kayamkulam 1 and Thilak showed the lowest levels (0.07%) of palmitoleic acid. A high 11.66% of stearic acid was found in *S. radiatum* oil, while Ayali 5 exhibited the lowest (6.06%). These findings align with Hiremath et al. (2007).

Eicosanoic acid was found only in Thilak among the cultivated varieties, while it was more prevalent in the traditional cultivar Ayali, particularly Ayali 4 (0.73%). 11-Eicosenoic acid was predominantly found in *S. malabaricum* (0.30%), while minimal amounts were found in both Thilak and Thilathara. *S. radiatum* contained the highest levels of linolelaidic acid, particularly in IC 256273. These findings contribute to the understanding of sesame fatty acid composition and offer valuable insights for sesame breeding programs focused on improving oil quality.

**Table 4** presents the mean values of morphological and biochemical traits in *Sesamum* species, highlighting key differences that can be used to effectively distinguish among them. These variations serve as important diagnostic features for species identification and have potential implications for breeding and conservation strategies.

## Conclusion

The analysis of variance performed on various morphological and biochemical parameters of *S. indicum*, *S. malabaricum*, *S. mulayanum*, and *S. radiatum* revealed significant differences, indicating substantial variability among the genotypes for the traits studied. All 27 sesame genotypes produced a single flower per axil, demonstrating that this characteristic is consistent across all sesame species. A comparative analysis of the different sesame species highlighted notable variations in the color of the lower lip of the flowers. The cultivated species *S. indicum* displayed pale purplish-pink flowers, while *S. mulayanum* and *S. malabaricum* had dark violet flowers, and *S. radiatum* exhibited white flowers with a violet border. Differences in capsule hairiness were also observed: cultivated species had glabrous capsules, while wild species produced hairy capsules, ranging from weakly hairy in *S. mulayanum* and *S. malabaricum* to strongly hairy in *S. radiatum*.

In terms of capsule production, the highest number of capsules per plant was found in Ayali 1, while *S. radiatum* (IC 256273) produced the fewest. *S. radiatum* accessions had the longest and broadest capsules, while *S. malabaricum* accessions produced the shortest and narrowest capsules. The cultivated varieties had heavier and larger seeds compared to the wild species.

Oil content analysis revealed the highest oil yield in Thilak seeds, while *S. malabaricum* (IC 557243) had the lowest oil yield. Fatty acid profiling of the four cultivated varieties and the wild relatives revealed the presence of both saturated fatty acids (palmitic, stearic, behenic, margaric, and arachidic acids) and unsaturated fatty acids (oleic, linoleic, linolenic, eicosanoic, 11-eicosenoic, and linolelaidic acids) in varying proportions. The highest phenol content was observed in *S. radiatum* (IC 210433), with the lowest in Kayamkulam 1. Lignin analysis revealed that sesamin content was highest in *S. malabaricum* (2.533), while sesamolin content was highest in *S. radiatum* (1.981). In *S. indicum* and *S. malabaricum*, sesamin constituted the major portion of lignin, whereas in *S. radiatum*, sesamolin content slightly exceeded that of sesamin.

## Data Availability

The original contributions presented in the study are included in the article/**Supplementary Material**. Further inquiries can be directed to the corresponding author.
