# Supplementary material for: Integrated morphological and biochemical analysis of selected sesame (Sesamum spp.) species
Source: Front Plant Sci. 2025 Jul 10;16:1571363. doi: 10.3389/fpls.2025.1571363 (PMC12287034; doi:10.3389/fpls.2025.1571363)
Supplement: Supplementary file 1 [file DataSheet1.pdf]

NIIST FAME ANALYSIS REPORT

Sample Information

Sample Name : Malabaricum
Sample ID : Malabaricum
Vial # : 2
Injection Volume : 1.00
$EndIf$Data File : G:\GCMS DATA\Ellu-N-24122022\Malabaricum_2.qgd
Org Data File : G:\GCMS DATA\Ellu-N-24122022\Malabaricum_2.qgd
Method File : G:\GCMS METHOD\LONG RUN FOR MORE COMPOUNDS - solovent cutoff 4 min.qgm
Org Method File : G:\GCMS METHOD\LONG RUN FOR MORE COMPOUNDS - solovent cutoff 4 min.qgm
Tuning File : G:\TUNING\ellui-n-24112022.qgt
If(!=)[Comment]

Chromatogram Malabaricum G:\GCMS DATA\Ellu-N-24122022\Malabaricum_2.qgd

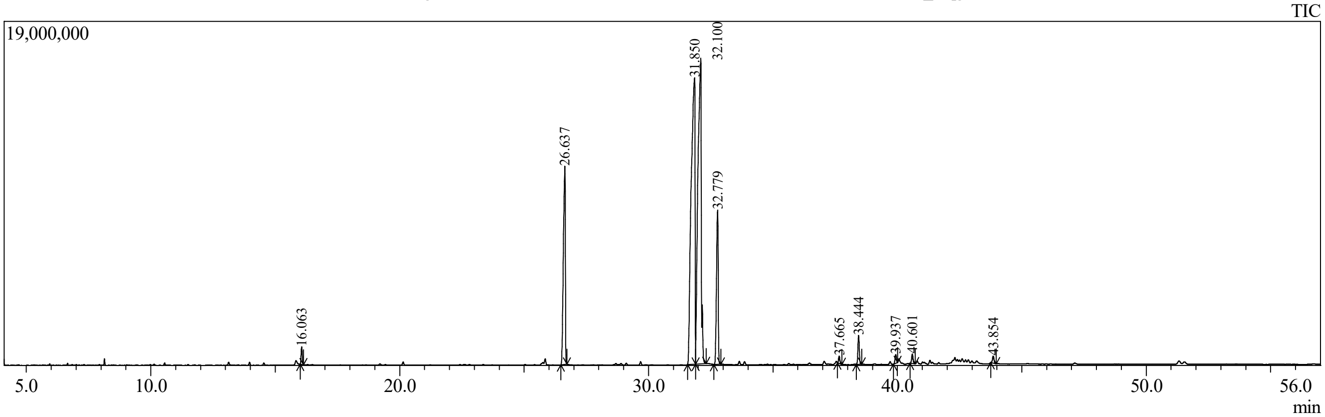

Peak Report TIC

Peak#	R.Time	Area	Area%	Name
1	16.063	3132647	0.70	Decanal dimethyl acetal
2	26.637	60272402	13.56	Methyl palmitate
3	31.850	168540217	37.93	Methyl linolelaidate
4	32.100	158386635	35.64	9-Octadecenoic acid, methyl ester, (E)-
5	32.779	40771979	9.18	Methyl stearate
6	37.665	1352903	0.30	11-Eicosenoic acid, methyl ester
7	38.444	6174376	1.39	Methyl arachisate
8	39.937	1977263	0.44	Methyl (11R,12R,13S)-(Z)-12,13-epoxy-11-m
9	40.601	2100796	0.47	Octadecanoic acid, 9,10-dihydroxy-, methyl es
10	43.854	1666433	0.38	Methyl behenate
		444375651	100.00	
