# Supplementary material for: Integrated morphological and biochemical analysis of selected sesame (Sesamum spp.) species
Source: Front Plant Sci. 2025 Jul 10;16:1571363. doi: 10.3389/fpls.2025.1571363 (PMC12287034; doi:10.3389/fpls.2025.1571363)
Supplement: Supplementary file 2 [file DataSheet2.pdf]

NIIST FAME ANALYSIS REPORT

Sample Information

Sample Name	: Mulayanam
Sample ID	: Mulayanam
Vial #	: 4
Injection Volume	: 1.00
$EndIf$Data File	: G:\GCMS DATA\Ellu-N-24122022\Mulayanam_4.qgd
Org Data File	: G:\GCMS DATA\Ellu-N-24122022\Mulayanam_4.qgd
Method File	: G:\GCMS METHOD\LONG RUN FOR MORE COMPOUNDS - solovent cutoff 4 min.qgm
Org Method File	: G:\GCMS METHOD\LONG RUN FOR MORE COMPOUNDS - solovent cutoff 4 min.qgm
Tuning File	: G:\TUNING\ellui-n-24112022.qgt
If(!=)[Comment]	

Chromatogram Mulayanam G:\GCMS DATA\Ellu-N-24122022\Mulayanam_4.qgd

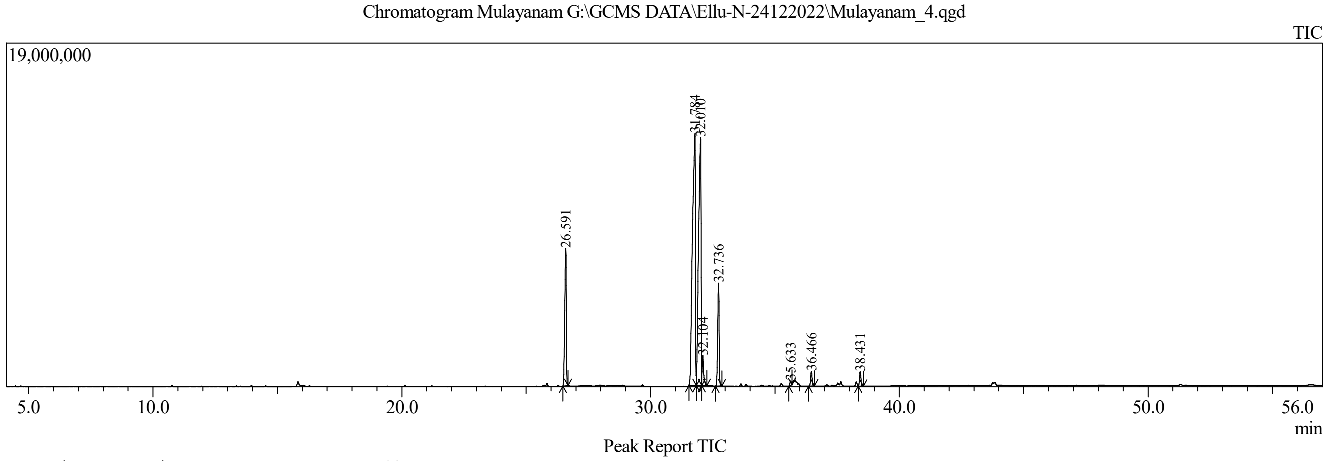

Peak Report TIC

Peak#	R.Time	Area	Area%	Name
1	26.591	33402912	11.93	Methyl palmitate
2	31.784	113511916	40.53	Methyl linolelaidate
3	32.010	95507286	34.11	9-Octadecenoic acid, methyl ester, (E)-
4	32.104	6870805	2.45	Methyl elaidate
5	32.736	23284959	8.31	Methyl stearate
6	35.633	881985	0.31	Methyl linolelaidate
7	36.466	3488127	1.25	9-Methoxytricosane
8	38.431	3089377	1.10	Methyl arachisate
		280037367	100.00	
