# Supplementary material for: Integrated morphological and biochemical analysis of selected sesame (Sesamum spp.) species
Source: Front Plant Sci. 2025 Jul 10;16:1571363. doi: 10.3389/fpls.2025.1571363 (PMC12287034; doi:10.3389/fpls.2025.1571363)
Supplement: Supplementary file 3 [file DataSheet3.pdf]

# NIIST FAME ANALYSIS REPORT

Sample Information

| | |
|---|---|
| Sample Name | : Radiatum |
| Sample ID | : Radiatum |
| Vial # | : 3 |
| Injection Volume | : 1.00 |
| $EndIf$Data File | : G:\GCMS DATA\Ellu-N-24122022\Radiatum_3.qgd |
| Org Data File | : G:\GCMS DATA\Ellu-N-24122022\Radiatum_3.qgd |
| Method File | : G:\GCMS METHOD\LONG RUN FOR MORE COMPOUNDS - solovent cutoff 4 min.qgm |
| Org Method File | : G:\GCMS METHOD\LONG RUN FOR MORE COMPOUNDS - solovent cutoff 4 min.qgm |
| Tuning File | : G:\TUNING\ellui-n-24112022.qgt |
| $If$(!=)[Comment] | |

Chromatogram Radiatum G:\GCMS DATA\Ellu-N-24122022\Radiatum_3.qgd

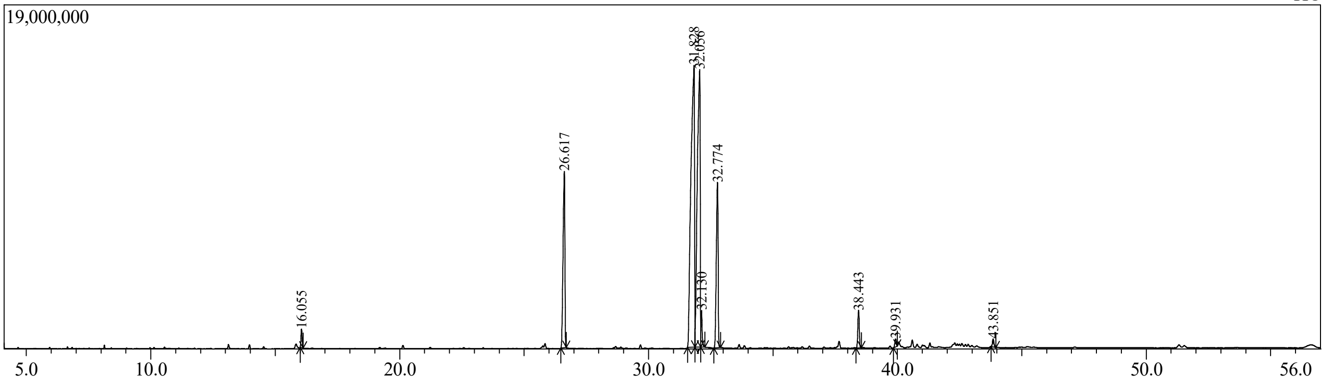


Peak Report TIC

| Peak# | R.Time | Area | Area% | Name |
|---|---|---|---|---|
| 1 | 16.055 | 3199589 | 0.82 | Decanal dimethyl acetal |
| 2 | 26.617 | 49072480 | 12.56 | Methyl palmitate |
| 3 | 31.828 | 152393079 | 39.00 | Methyl linolelaidate |
| 4 | 32.056 | 122433107 | 31.34 | 9-Octadecenoic acid, methyl ester, (E)- |
| 5 | 32.130 | 6117250 | 1.57 | Methyl elaidate |
| 6 | 32.774 | 45567220 | 11.66 | Methyl stearate |
| 7 | 38.443 | 8324553 | 2.13 | Methyl arachisate |
| 8 | 39.931 | 1833563 | 0.47 | Methyl (11R,12R,13S)-(Z)-12,13-epoxy-11-m |
| 9 | 43.851 | 1777221 | 0.45 | Methyl behenate |
| | | 390718062 | 100.00 | |
