# Supplementary material for: Integrated morphological and biochemical analysis of selected sesame (Sesamum spp.) species
Source: Front Plant Sci. 2025 Jul 10;16:1571363. doi: 10.3389/fpls.2025.1571363 (PMC12287034; doi:10.3389/fpls.2025.1571363)
Supplement: Supplementary file 4 [file Table1.docx]

**Supplementary table 1. Details of the plant materials used in the study**

| Sl. No. | Species | Accession number/Variety | Biological Status | Place of collection |
| --- | --- | --- | --- | --- |
| 1. | *Sesamum indicum* | Kayamkulam 1 | Variety | Kerala Agricultural University |
| 2. |  | Thilak | Variety |  |
| 3. |  | Thilathara | Variety |  |
| 4. |  | Thilarani | Variety |  |
| 5. |  | Ayali 1 | Cultivar | Alappuzha, Kerala |
| 6. |  | Ayali 2 | Cultivar | Alappuzha, Kerala |
| 7. |  | Ayali 4 | Cultivar | Alappuzha, Kerala |
| 8. |  | Ayali 5 | Cultivar | Alappuzha, Kerala |
| 9. |  | Ayali 11 | Cultivar | Alappuzha, Kerala |
| 10. | *Sesamum mulayanam* | IC 557231 | Wild | Satara, Maharashtra |
| 11. |  | IC 277406 | Wild | Thrissur, Kerala |
| 12. |  | IC 277417-X | Wild | Kannur, Kerala |
| 13. |  | IC 557232 | Wild | Satara, Maharashtra |
| 14. |  | IC 199447 | Wild | Thrissur, Kerala |
| 15. |  | SML 1 | Wild | Alappuzha, Kerala |
| 16. |  | SML 2 | Wild | Alappuzha, Kerala |
| 17. | *Sesamum malabaricum* | IC 557251 | Wild | Raigad, Maharashtra |
| 18. |  | IC 621506 | Wild | Ratnagiri,Maharashtra |
| 19. |  | IC 557244 | Wild | Raigad, Maharashtra |
| 20. |  | IC 623409 | Wild | Navsari,Gujarat |
| 21. |  | IC 557243 | Wild | Raigad, Maharashtra |
| 22. | *Sesamum radiatum* | IC 210433 | Wild | Thrissur, Kerala |
| 23. |  | IC 623402 | Wild | Kannur, Kerala |
| 24. |  | IC 208681 | Wild | Alappuzha, Kerala |
| 25. |  | IC 256273 | Wild | Kerala |
| 26. |  | IC 208663 | Wild | Thrissur, Kerala |
| 27. |  | SR 1 | Wild | Alappuzha, Kerala |
